# Risk Factors Associated With Renal and Urinary Tract Anomalies Delineated by an Ultrasound Screening Program in Infants

**DOI:** 10.3389/fped.2021.728548

**Published:** 2022-01-24

**Authors:** Yuling Liu, Hua Shi, Xiaojing Yu, Tianchao Xiang, Ye Fang, Xian Xie, Xiaofen Pan, Xiaolin Li, Zhicai Sun, Bihong Zhang, Simao Fu, Jia Rao

**Affiliations:** ^1^Department of Pediatrics, Boai Hospital of Zhongshan Affiliated to Southern Medical University, Zhongshan, China; ^2^Department of Nephrology, Children's Hospital of Fudan University, National Children's Medical Center, Shanghai, China; ^3^Shanghai Kidney Development and Pediatric Kidney Disease Research Center, Shanghai, China; ^4^Shanghai Key Lab of Birth Defect, Children's Hospital of Fudan University, Shanghai, China

**Keywords:** congenital anomalies of the kidney and urinary tract (CAKUT), decision curve analysis (DCA), predictive model, risk factors, urinary tract dilation (UTD)

## Abstract

**Objective:**

To evaluate the value of ultrasound screening for congenital anomalies of the kidney and urinary tract (CAKUT) during the early postnatal period.

**Methods:**

This is a prospective study that enrolled all neonates born from August 2019 to July 2020 at one medical center. Postnatal ultrasound screening was conducted in all neonates at 1, 3, and 6 months old, respectively. Information on antenatal detection and pregnancy was collected. We performed logistic regression analyses and established a predictive model to assess the potential risk factors of abnormal ultrasound screening results.

**Results:**

Postnatal ultrasound scanning in 4,877 infants identified 268 cases (5.5%) of anomalies of kidney and urinary tract by primary screening and 92 cases (1.9%) by tertiary screening. A specific diagnosis was identified in 47 cases within the 6-month screening and follow-up program. Logistic regression revealed that preterm birth, oligohydramnios, antenatal ultrasound screening anomalies, and gestational hypothyroidism were independent risk factors for the early detection of CAKUT by postnatal ultrasound screening. The above factors were adopted to develop a predictive model that showed good calibration in predicting ultrasound findings of CAKUT. Decision curve analysis demonstrated good clinical utility.

**Conclusions:**

Postnatal ultrasound screening should be conducted in infants with risk factors associated with CAKUT. Further study on prenatal and fetal factors could help establish the predictive model for the early detection of CAKUT.

## Introduction

Congenital anomalies of the kidney and urinary tract (CAKUT) account for 10–20% of all major congenital anomalies (1). It is a significant cause of morbidity and mortality in neonates (2) and is also the main cause in children and adolescents with end-stage renal disease (ESRD) (1, 3). Renal dysfunction often progressed by the time without any symptoms or abnormal findings by urinalysis for children with CAKUT. The advancing technology in medical equipment improves the detection of kidney and urinary tract anomalies *in utero* by prenatal ultrasonography. However, prenatal ultrasonographic diagnosis may fail to detect these congenital anomalies in oligoamnios and other rare situations (4). Lots of attempts have been made for CAKUT screening during the early postnatal period (5–8). The current study presents such screening in 1-month-old infants and follow-up in 6 months conducted in the medical centers in Zhongshan City, China. At the end of 2020, Zhongshan had a permanent population of 4.41 million with the birth rate of 12.4 per 1,000 population. In terms of age distribution, 15.6% was aged 0–14 years covering 0.02% of China's population aged 0–14 years (http://www.stats.gov.cn/). The specific aim of this study was to explore the potential risk factors associated with abnormal ultrasound screening results, which could help to establish the population with a high risk of CAKUT by postnatal ultrasound screening.

## Methods

### Setting and Participants

This study was a prospective study that consecutively enrolled all neonates born from August 2019 to July 2020 in the Zhongshan Boai Hospital in which one-fourth of newborns in the city are provided medical care. We solicited the records in medical charts for pregnancy including the medical history of the mother, pregnancy and delivery, family history, as well as the results of several postnatal examinations of the newborns (i.e., screening for inherited metabolic disorders by dried-blood spot specimens, ultrasound screening of the kidneys and the hips, and audiometry screening). The study design and data management were approved by the local ethics committee (KY-2019-002-17), and written informed consent was obtained from all parents or legal guardians prior to the inclusion of the infants in the study. During the hospital stay, the study after delivery, the process of conducting renal ultrasonographic screening was explained in writing, and an interview sheet was provided to the parents or guardians. Medical charts for pregnancy including maternal and fetus information were subsequently recorded upon written consent. Most of the children performed postnatal ultrasound screening initially at the 1-month checkup. Urgent postnatal ultrasound sonography was performed within the first postnatal week in cases with abnormal antenatal ultrasonic findings. Exclusion criteria were missing consent, stillbirth, incomplete records, and loss of follow-up in 6 months. As a routine part of caring for pregnant women, prenatal screening commonly consists of at least two examinations during pregnancy, one of which is 20–24 weeks anatomy scan and the other one is conducted in 30–33 weeks. The information of antenatal ultrasound findings for kidney was solicited from routine screening in both of them.

### Ultrasound Measurements

Ultrasound tests were conducted by ultrasonographers certified by the health administrative department post more than 5 years of practice. A GE Voluson 730 scanner (GE Healthcare, Little Chalfont, UK) with a 5–7 MHz convex-type transducer was used to screen all the participants. Recordings consisted of at least 6 bilateral renal images, including one longitudinal and two transverse images obtained in the supine position. The urinary bladder was scanned in the supine position. The image was recorded only when an abnormality was identified. No feeding restriction was imposed, and no sedative was administered before screening.

The following features of kidneys were assessed: presence vs. absence, malposition, sizes, and differences between left and right sides, the separation of the central echo complex, renal echogenicity, assessment of the renal cortex-medullar differentiation, and other abnormalities such as cysts or tumors. Abnormalities in bladder shape and wall, as well as retrovesical ureteral dilation were checked. If a dilation of the renal pelvis or/and retrovesical ureteral dilation is detected, the abdominal way from renal pelvis to bladder should be screened for megaureter, which might be meandering. Criteria for abnormalities (7, 9–12) included kidney length according to the reference for preterm or term infants; a difference of ≥10 mm in length of the left and right kidneys; and if urinary tract dilation (UTD) was detected, we used the Onen hydronephrosis-grading system (10) combined with caliectasis, parenchymal thinning and the anteroposterior diameter of the renal pelvis (APD).

### Follow-Up and Outcome

**Figure 1:**
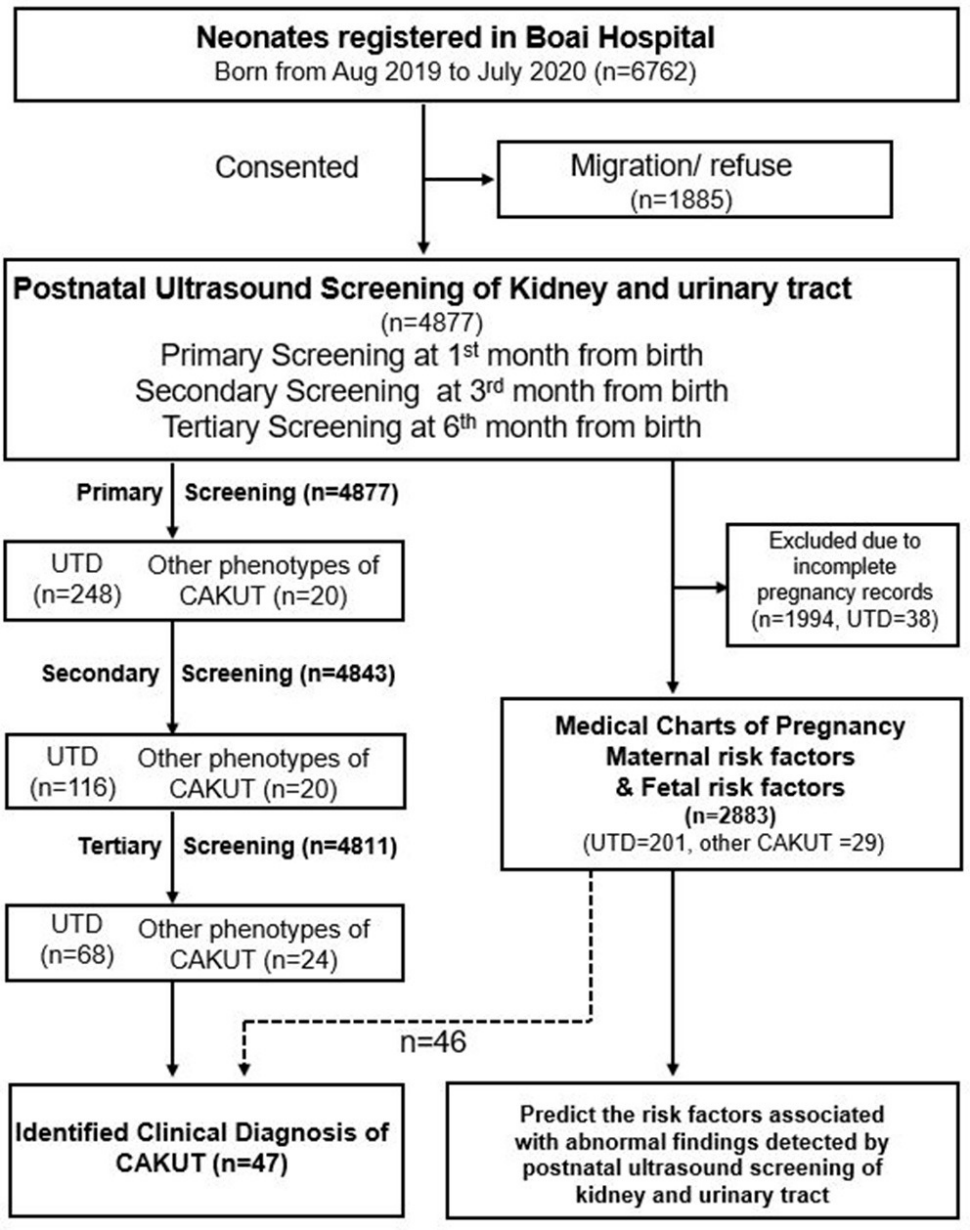
Flowcharts of study participants. CAKUT, congenital anomalies of the kidney and urinary tract; UTD, urinary tract dilation.

All the children underwent the secondary screening within the 3rd month and the tertiary screening during the 6th month post primary screening (Figure 1). Parents were given a review of the results when ultrasonography showed abnormal findings. We recommended further examination to the infants with persistent abnormal findings of ultrasound scanning during the follow-up period.

In the cases of UTD, we categorized three groups of cases according to the measurement of the renal pelvis: (i) APD < 5 mm, no special measures, routine care; (ii) APD ≥ 5 mm and <10 mm, detailed ultrasound examination of the urinary tract by a pediatric radiologist after 1–2 months, an individualized approach based on the findings; and (iii) moderate/sever: APD ≥ 10 mm, or caliceal dilation confirmed by a detailed ultrasound examination by a pediatric radiologist, an individualized diagnostic and therapeutic approach, surveillance by a pediatric nephrologist.

During the follow-up, we defined a complete remission, partial remission, stable, progressive, and complications as follows: (i) complete remission: APD < 5mm; (ii) partial remission: APD was smaller; (iii) stable: APD was unchanged; (iv) progressive: APD was larger; and (v) complications: other types of CAKUT, UTI (13), and calculi were discovered.

### Data Collection and Statistical Analysis

Enumeration data were presented as median (P25, P75) and rate. The proportion of abnormal findings was calculated in the study population. We estimated the risk factors including preterm birth, low birth weight children, macrosomia, *in vitro* fertilization-embryo transfer (IVF-ET), antenatal detection of kidney anomalies, congenital heart disease or fetal chromosome abnormality, oligohydramnios, preeclampsia (including early preeclampsia and preterm preeclampsia), gestational diabetes, gestational hypertension, hypothyroidism (14), hyperthyroidism, or infection during pregnancy. Univariate analysis was initially performed to determine the possible correlation between potential risk factors and abnormal postnatal findings on CAKUT. Associations were estimated by the odds ratio (OR) and corresponding 95% confidence intervals (CIs). All significant risk factors (*p* < 0.1) in the univariate logistic regression analysis were eligible for inclusion in the multivariate logistic regression analysis to adjust for confounding factors. Thereafter, we assessed the statistical interaction between the confounding factors. Multivariate model #1 was applied to all variables without complete separation. Multivariate model #2 was applied to the confounding variables with a *p* < 0.1. Based on the multivariate logistic regression model, a simplified nomogram was constructed with statistical packages R (14). The Harrell's concordance index (C-index) was performed to quantify the discrimination performance of the simplified nomogram. The performance of the nomogram model was assessed using its calibration and discrimination. Calibration describes the level of agreement between the predicted and actual risks, and is usually evaluated by a calibration plot and Hosmer–Lemeshow χ^2^-test. Discrimination refers to the ability of a model to distinguish participants in model#1 and participants in model#2, and is generally evaluated by C-index or area under the receiver operator characteristic curve (AUROC). Decision curve analysis (DCA) was performed to evaluate the clinical usefulness of the nomogram model by quantifying the net benefits under different threshold probabilities. All statistical analyses were performed on GraphPad Prism 9 (GraphPad Software, La Jolla, California, USA) and statistical packages R version 3.6.1 (R Development Core Team). The packages of rms, Hmisc, pROC, stats, PredictABEL, and rmda were involved in this process.

## Results

A total of 4,877 newborns participated in this study, accounting for 72.1% (4,877/6,762) of all births in Zhongshan City from August 2019 to July 2020. None of the birth records were homebirth or unskilled birth attendants. Of these, 4,811 (98.6%) cases (M:F ratio = 1.1:1) had complete screening records and follow-up information (Figure 1).

### Presentation of Malformations During Screening

We initially screened 4,877 infants, detecting 268 (5.5%) cases with abnormal findings. The most prevalent abnormal finding was UTD (*n* = 248), followed by duplex kidney (*n* = 7), abnormal size or location of kidney (*n* = 5), renal cysts (*n* = 3), and abnormal ureter and bladder (*n* = 5). Subsequently, the tertiary screening detected 92 (1.9%) cases with remaining abnormal findings, including additional cases of renal calcinosis or nephrolith (*n* = 4). Among the 4,671 neonates with full records of antenatal ultrasound, CAKUT had been detected prenatally in 91 (1.9%) fetuses including one case of unilateral renal agenesis and 90 cases of antenatal hydronephrosis. Suspicion of CAKUT by antenatal ultrasound was confirmed postnatally in 42.9% (39/91) of infants including 35 cases with unilateral UTD, 3 cases with bilateral UTD, and one case with unilateral renal agenesis. Persistent UTD was recorded in the 38 cases during the 6-month-period of follow-up. Newly identified abnormalities were detected in 229 infants through postnatal ultrasound screening including 210 cases of UTD and 19 cases of other types of CAKUT.

Among the 248 cases with UTD, bilateral dilation accounted for 19.4% (48/248), UTD on the left side accounted for 66.6% (166/248) and UTD on the right side accounted for 13.7% (34/248). Hydronephrosis involved in at least one kidney presented in 232 cases with Onen grade 1 (213 with APD < 5 mm; 19 with APD < 5–10 mm) and 16 cases with Onen grade 2 (Supplementary Table 1).

**Figure 2:**
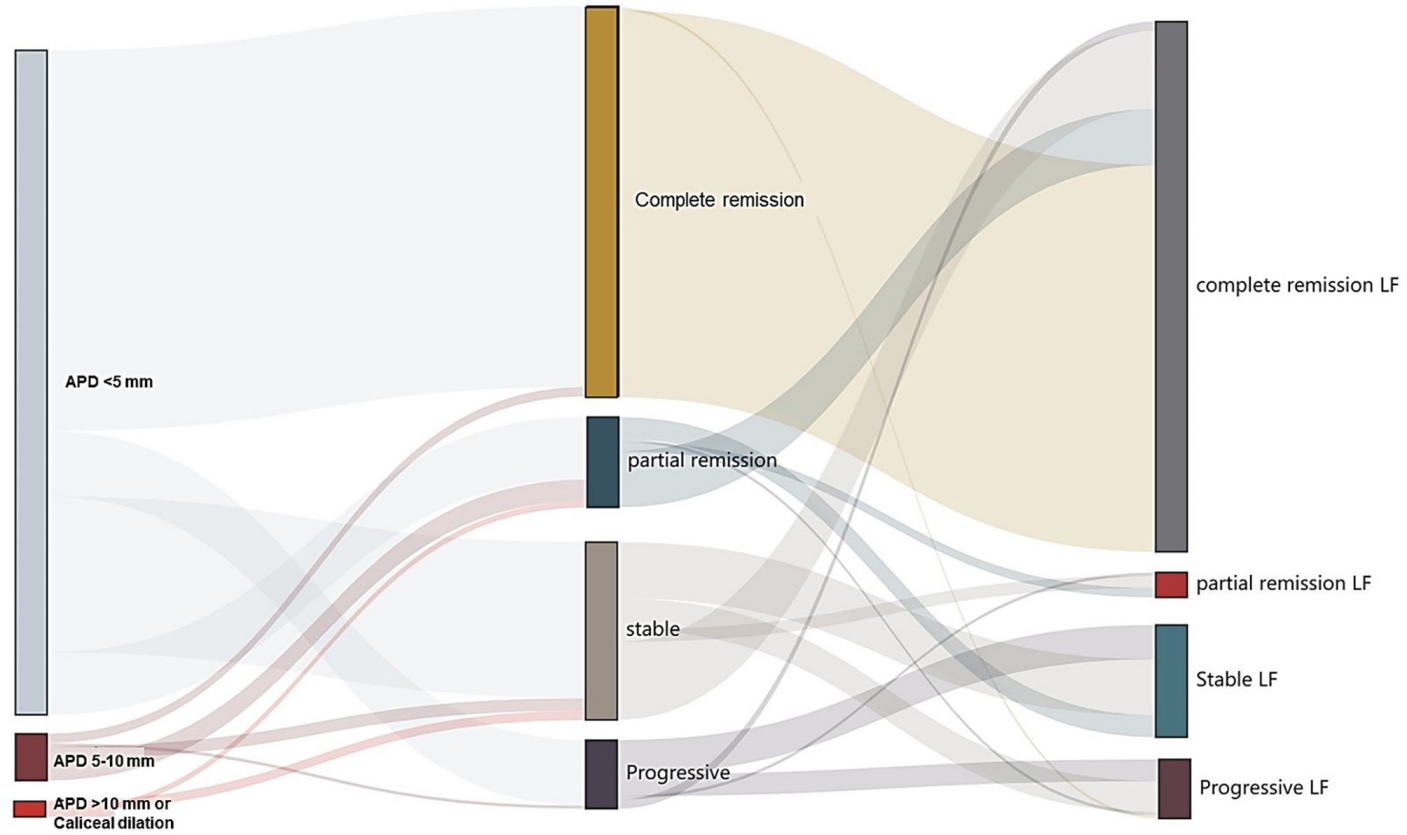
Follow-up of children with urinary tract dilation (UTD). Left and middle: Division of the primary and secondary ultrasound screening results. Middle and right: Division of change in secondary and tertiary ultrasound screening results. The width of the lines in the Sankey plot is proportional to the relative quantity of cases within each group. APD, the anteroposterior diameter of the renal pelvis; LF, the last follow-up.

During the 6-month follow-up, 98.6% (4,811/4,877) of participants complete the secondary and tertiary screening or further examinations. After the secondary screening at the 3rd month follow-up, the resolution of UTD was seen in 132 (53.3%) participants, partial remission was seen in 37 participants, remaining (or stable) was seen in 61 participants, progressive UTD was seen in 18 participants, and 7 episodes of urinary tract infection were reported. After the third screening at 6th month follow-up, the resolution of UTD was seen in 180 (72.6%) participants, partial remission was seen in 20 participants, remaining (or stable) was seen in 44 participants, progressive UTD was seen in 4 participants, and additional 15 episodes of urinary tract infection were reported (Figure 2 and Supplementary Table 2). Additionally, there were 15 participants with normal antenatal screening and normal postnatal ultrasound initially who were detected of UTD with APD < 5 mm during the follow-up period of 3rd to 6th month. There was no case of unilateral UTD developed into bilateral UTD. Among the 19 patients with episodes of afebrile UTI, three patients had recurrent UTI. There were no participants with abnormal renal function based on the records of estimated GFR by Schwartz formula. Transient renal medullary hyperechogenicity (unilateral/bilateral) was reported in 11 of 134 preterm neonates without any other abnormal findings of ultrasound or any clinical evidence of kidney damage during the first week post-delivery. Follow-up scan during the 3rd to 6th month showed that the medullary changes had resolved completely in the 11 cases.

There were 47 patients (1.0%) who received a specific diagnosis (Table 1). The most frequently encountered was obstructive uropathy [ureteropelvic junction obstruction (UPJO), *n* = 15], followed by vesicoureteral reflux (VUR, *n* = 7), duplex kidney (*n* = 4), and renal agenesis/dysplasia (*n* = 5). All diagnosed UPJO occurred in the patient group with UTD Onen grade 2. All cases with VUR came from the patient group with UTD Onen grade 1 (APD < 10 mm) who was found by VCUG following the episode of afebrile UTI. Bilateral VUR was found in four cases (grade IV in 2 ureters, grade III in 1 ureter, grade II in 2 ureters, and grade I in 1 ureter). Unilateral VUR was identified in three cases of grade III. No abnormal findings of renal size or renal function were reported in the seven cases of VUR. Out of the 15 children with UPJO, 12 patients underwent surgery within 1 year. Among the 47 cases with a specific diagnosis of CAKUT, abnormal prenatal ultrasound findings were detected in 22 cases (Table 1).

**Table 1:** Specific disorders found in examination of 47 cases following ultrasonographic screening.

**Types**	**Number of patients (*n* = 47)**	**Cases with abnormal antenatal ultrasound findings (*n* = 22)**	**Male/female**
Ureteropelvic junction obstruction	15	12	11/4
Vesicoureteral reflux	7	0	5/2
Duplex kidney Nephrolith or renal calcinosis	4 4	4 0	2/2 3/1
Renal agenesis	3	2	1/2
Ureteral cyst and duplex kidney	3	1	0/3
Renal cyst Bladder cyst Ectopic pelvic kidney Megaureter The posterior urethral valve Ureteral terminal cyst Renal dysplasia Ectopic and dysplasia kidney	3 2 1 1 1 1 1 1	0 0 0 1 1 0 0 1	1/2 2/0 0/1 1/0 1/0 1/0 0/1 0/1

### Risk Factors Associated With Abnormal Findings of Ultrasound Screening

We evaluated the OR values for the potential risk factors reported in pregnancy charts and fetus information between the participant groups with or without abnormal findings through postnatal ultrasound screening. For the reason of incomplete record of pregnancy charts, 1,994 participants including 38 cases with UTD were not enrolled in the risk factor analysis. Among the 2,883 participants with complete pregnancy medical chart, abnormalities were detected in 230 cases through postnatal ultrasonography (Figure 1). A combined analysis of all potential risk factors showed that children with one of the risk factors had a significantly higher odds ratio for postnatal abnormal findings (OR, 1.64; 95% CI, 1.20–2.22) than those without any prenatal risk factors. Logistic regression analysis was conducted to determine the set of variables that jointly and independently predict postnatal ultrasound screening for CAKUT. The adjusted odds ratios that were statistically significant predictors of the postnatal ultrasound outcomes were gender, preterm birth, oligohydramnios, antenatal ultrasound screening anomalies, and gestational hypothyroidism (Table 2). Statistically significant interactions were found between gestational hypothyroidism (yes or no) on offspring preterm birth (*p* < 0.001). Thereafter, the adjusted association between the identified risk factors and CAKUT detection was documented in the 2,749 term neonates (Table 3). For example, mothers with hypothyroidism had a 15-fold higher risk of having a neonate with CAKUT (adjusted OR,14.5). The characteristics of infants with abnormal screening results were similar to those without abnormal screening results with respect to birth weight, IVF-ET, and maternal diabetes/hypertension/infection during pregnancy. These factors did not add significant incremental information over and above those four risk factors (Table 2). There were only a few cases reported with fetal heart deficiency, intrauterine infection, severe preeclampsia, hyperthyroidism during pregnancy, polyhydramnios, maternal CAKUT, chromosomal abnormalities in amniocentesis, or polycystic ovary syndrome, which were excluded from the logistic analysis due to complete separation.

**Table 2:** Univariate and multivariate logistic regression analysis of potential risk factors associated with kidney and urinary tract anomalies in 2,883 participants screened by postnatal ultrasound scanning.

**Risk factors**	**Abnormal findings by postnatal ultrasound screening**	**No abnormal findings by postnatal ultrasound screening**	**Univariate analysis**		**Logistic regression analysis**	
	**(*n* = 239)**	**(*n* = 2,644)**	**OR (95% CI)**	***p*-value**	**β regression coefficient**	**Standard error (95% CI)**
**Gender**					2.1	0.2 (25, 1.6)
Female	84	1,228	1.0 (Ref)			
Male	155	1,416	1.6 (1.2, 2.1)	0.0009	0.4	0.1 (0.1, 0.7)
**Preterm**						
No	220	2,529	1.0 (Ref)			
Yes	19	115	1.9(1.1, 3.1)	0.0113	0.8	0.3 (0.2, 1.4)
**Macrosomia**						
No	200	2,592	1.0 (Ref)			
Yes	39	52	9.7 (6.2, 15.2)	<0.0001	0.3	0.3 (-0.4, 1.0)
**Low birth weight**						
No	228	2,534	1.0 (Ref)			
Yes	11	110	1.1 (0.6, 2.1)	0.7354		
**IVF-ET**						
No	235	2,409	1.0 (Ref)			
Yes	18	221	0.8 (0.5, 1.4)	0.4778		
**Antenatal ultrasound detection of kidney or urinary tract anomalies**						
No	200	2,592	1.0 (Ref)			
Yes	39	52	9.7 (6.2, 15.2)	<0.0001	2.3	0.2 (1.8, 2.7)
**Gestational diabetes**						
No	184	2,034	1.0 (Ref)			
Yes	55	610	1.8 (0.7, 1.4)	0.02058	0.1	0.2 (-0.4, 0.3)
**Hypothyroidism**						
No	237	2,642	1.0 (Ref)			
Yes	2	2	0.2 (0.1, 0.6)	0.0367	2.7	1.0 (0.6, 4.8)
**Oligohydramnios**						
No	233	2,624	1.0 (Ref)			
Yes	6	20	3.4 (1.4, 8.4)	0.006	1.3	0.5 (0.3, 2.2)
**Preeclampsia**						
No	230	2,575	1.0 (Ref)			
Yes	9	69	1.5 (0.7, 2.9)	0.2915		
**Gestational hypertension**						
No	229	2,550	1.0 (Ref)			
Yes	10	94	1.2 (0.6 to 2.2)	0.6176		
**Gestational infection**						
No	225	2,528	1.0 (Ref)			
Yes	14	116	1.4 (0.8, 2.4)	0.294		

*Univariate and multivariate logistic regression analysis of potential risk factors associated with kidney and urinary tract anomalies in 2,883 participants with complete information of pregnancy medical charts. Fertilization-embryo transfer, IVF-ET*.

**Table 3:** Association between the identified risk factors and kidney and urinary tract anomalies screened by postnatal ultrasound scanning in full-term neonates.

**Risk factors**	**Univariate analysis**			**Logistic regression analysis**	
	**Crude OR (95% CI)**	***p*-value**	**β**	**Standard error (95% CI)**	**Adjusted OR (95% CI)**
**Gender**					
Female	1.0 (Ref)				
Male	1.8 (1.3, 2.5)	<0.0001	0.6	0.5 (0.2, 0.8)	1.7 (1.3, 2.3)
**Antenatal ultrasound detection of kidney or urinary tract anomalies**					
No	1.0 (Ref)				
Yes	0.1 (0.1, 1.1)	<0.0001	2.4	2.3 (1.9, 2.8)	10.4 (8.7, 16.4)
**Hypothyroidism**					
No	1.0 (Ref)				
Yes	11.6 (0.1, 2.9)	<0.0001	2.5	2,7 (0.7, 4.6)	14.5 (2.0, 108.2)
**Oligohydramnios**					
No	1.0 (Ref)				
Yes	0.3 (0.1, 0.8)	0.021	1.2	1.3 (0.3, 2.3)	3.6 (1.3,10.0)

**Figure 3:**
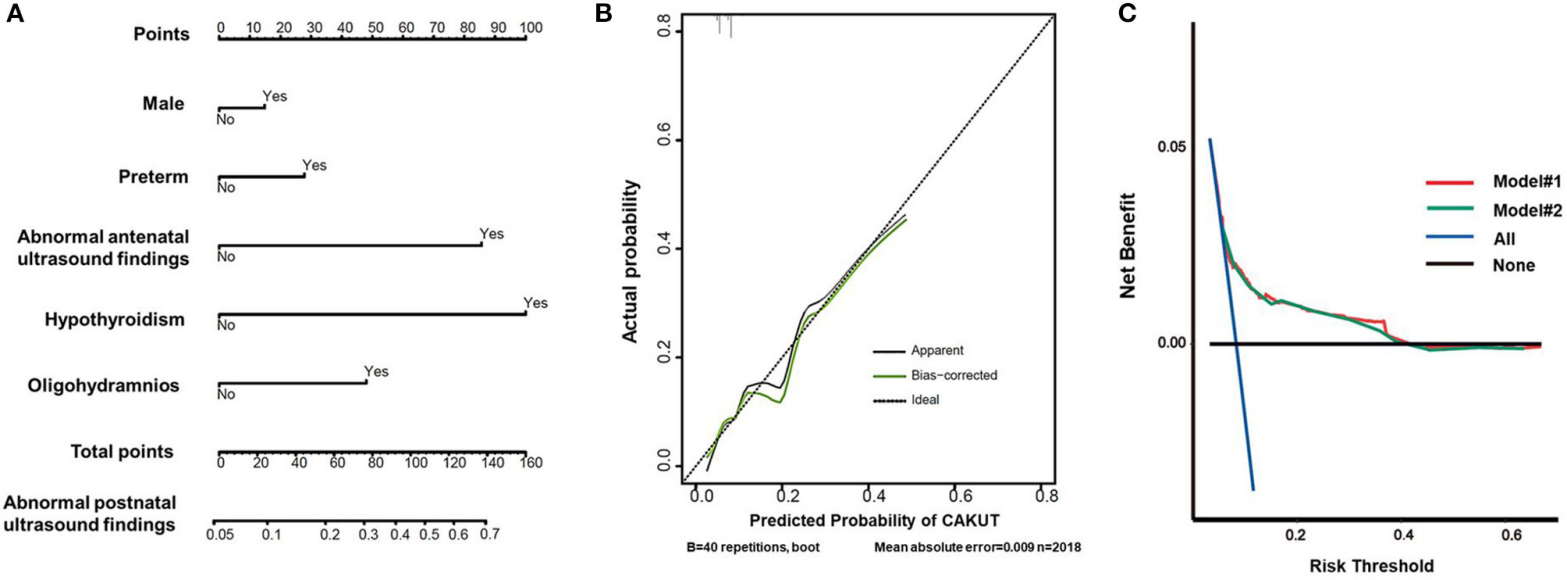
Prediction of abnormal findings by postnatal ultrasound screening for CAKUT. **(A)** Nomogram predicting postnatal ultrasound screening result. To utilize the nomogram, an individual patient's value was presented on each variable axis, and the vertical line was down upward to find the number of points received for each variable value. The sum of the variable values was presented on the total point axis and a vertical line was drawn downward to the probabilities of abnormal screening outcome. **(B)** Calibration curve of the predictive model. The *x*-axis showed the predicted abnormal screening results. The *y*-axis showed the actual abnormal screening results. The vertical lines show the frequency distribution of the predicted positive ratio of screening result. The apparent calibration curve (dotted line) indicates the model performance in the original data, while the bias-corrected curve (solid line) represents the model performance after optimism correction using 1,000 bootstrapped resamples. A perfect prediction would fall on the 45-degree (dashed) reference line. **(C)** Decision curves for the nomogram model. The *y*-axis measures the net benefit and the *x*-axis shows the threshold probability. The horizontal black line along the *x*-axis represents the assumption that no infants will perform postnatal ultrasound screening, whereas the solid aqua line represents the assumption that all infants will perform postnatal ultrasound screening. The red line indicated the nomogram model #1 in participants with the 4 propounding risk factors. The green line indicated the nomogram model #2 in participants with the 12 risk factors.

Based on the multivariate logistic regression analysis, a nomogram prediction model was constructed by integrating the independent risk factors (Figure 3A). Each factor was ascribed a weighted point, and the total points indicated the risk of CAKUT detection by postnatal ultrasonography. The Kattan-style nomogram in Figure 3A is the graphical representation of the logistic regression formula. A vertical line drawn from the scale value of each predictor down to the “Score” scale provides the numerical score for that predictor. The sum of the predictors yields the “Total Score,” which can be scaled to the final output probability of CAKUT detection. For example, a male infant was associated with 15 points, preterm birth was associated with 30 points, and a mother with hypothyroidism was associated with 100 points, yielding a total score of points 145 points. This score indicated that this patient had a 70% risk of CAKUT detected by ultrasound postnatally. Our nomogram prediction model showed a close correspondence with the actual status of the screening results, as shown in the calibration curve (Figure 3B). To assess the accuracy and clinical application potential, we introduced the C-index and DCA. The C-index of our nomogram model#2 was 0.656 (95% CI 0.618 to 0.695), which did not show a significant difference compared with the C-index (0.659, 95% CI 0.620–0.698) of model#1 (*p* > 0.05). Model calibration was evaluated using the Hosmer–Lemeshow χ^2^-test, which was 2.337 (*p* = 0.505), demonstrating that there was no significant difference from a perfect fit. The DCA indicated that when the threshold probability is within a range of 0.05 to 0.4, use of the nomogram for predicting CAKUT would provide greater benefit than the “treat all (standard operation)” or “treat none (local excision)” regimen. With several overlap, the net benefit of the nomogram model#1 was comparable to that of model#2 (Figure 3C).

## Discussion

In the current study, we presented the results of postnatal ultrasound screening for CAKUT in a total of 4,877 newborns in one medical center. Postnatal ultrasound detected CAKUT in 5.5% newborns by primary screening and 1.9% by tertiary screening. A specific diagnosis was identified in 47 cases during the 6-month screening and follow-up program. Furthermore, risk factors of maternal hypothyroidism and other factors of fetus were identified to be associated with the abnormal findings of postnatal ultrasound screening for CAKUT.

CAKUT comprises a wide spectrum of defects that range from asymptomatic abnormalities to lethal conditions (2). Early detection of CAKUT relies upon a screening program among fetuses, neonates, and infants (15). Although there is little doubt on the utility of ultrasound for early detection of CAKUT, there is some controversy on timing, population for screening, and cost burden associated with using ultrasound as a screening test (8, 15). Based on this prospective study, we assessed the risk factors for CAKUT. Male gender, preterm birth, antenatal abnormal ultrasound findings, gestational hypothyroidism, and oligohydramnios were associated with higher risks of CAKUT detection. However, birth weight, IVF-ET, maternal diabetes or hypertension, and infection during pregnancy were not useful predictors in this study. The WHO estimates the increasing preterm birth rates. It has been shown that the pooled preterm birth rate in China was 6.09% (16). Preterm birth rate varied by regions. In our study, the ratio of preterm birth was 4.6%, which was comparable with the preterm birth incidence of the neighboring cities (17). Considering the known association of maternal hypothyroidism and preterm birth (18), we evaluated the risk factors associated with CAKUT in term infants. Gestational hypothyroidism was found to be a significant risk factor for offspring CAKUT either in preterm infants or in term infants. However, only four cases with gestational hypothyroidism were involved in our study. The expected incidence of gestational hypothyroidism ranges from 0.3 to 11% depending on definition and geographical area (14). No large study has shown, to our knowledge, the association of maternal hypothyroidism with offspring CAKUT. Our results would add to the limited knowledge on the multifactorial mechanisms underlying CAKUT.

Nomograms represent a novel model to provide predictive information tailored to the individual, based on the risk factors that exhibited significant differences in multivariate analysis (15). Nomograms are increasingly being applied to clinical decision-making. Currently, in the field of CAKUT, most of the studies have shown the risk factors of CAKUT with the application of logistic regression but lack of a prediction model. Here, we built the nomogram for predicting CAKUT using the new statistical method in R language. From the logistic regression shown in Table 2, one might conclude that male gender (OR 1.5), preterm (OR 2.1), and oligohydramnios (OR 3.6) would not contribute much to the final risk score compared to antenatal ultrasound screening abnormalities (OR 9.9). By using the nomograms for a male (15 points) preterm baby (30 points) who was born to a mother with oligohydramnios (50 points), the total 95 points correspond to a 60% risk of CAKUT detected by ultrasound postnatally. However, a female term baby with abnormal antenatal findings (90 points) yields a total score of 90 points, which indicates only a 55% risk of CAKUT detection postnatally. Hence, nomograms can extract as much information as possible from clinical data to provide the most accurate predictions from clinical data. Comparing the C-index and the ROC curve for the two models involved in 12 or 4 risk factors, respectively, provided no comparable discriminating power. As the C-index of nomogram prediction model was only 0.656, further studies on a larger cohort are needed to optimize the prediction model. With benefits of easy application and no need for additional information collection, the DCA was widely used to evaluate the clinical prediction models. In the current study, DCA demonstrated that the clinical net benefit of the nomogram was larger than that in the hypothetical all-screening or non-screening scenarios for a wide range of thresholds (5–40%). Therefore, our nomogram prediction model could be a useful tool to estimate the clinical risks of CAKUT for neonates and infants. It would provide an avenue for targeted screening identified the children with a high risk of CAKUT.

Antenatal ultrasounds can help to diagnose CAKUT in 60–85% of infants, especially if imaging is performed in the third trimester (19, 20). Prenatal ultrasound alone was found to have a relatively low sensitivity and a high specificity for detecting CAKUT. In this study, abnormal results of antenatal ultrasound screening were confirmed in 43% of the neonates by postnatal ultrasound and 85% (229/268) of abnormalities were detected through postnatal screening. It may indicate the poor quality of the antenatal ultrasound for kidneys. Prenatal screening, as a routine part of caring for pregnant women, is not specifically focused on the urinary tract, neither are standardized clinical techniques. Moreover, it is often carried out by inadequately trained professionals using equipment of varied quality. Hence, more attention should be paid to qualified prenatal screening for kidney and urinary tract.

Recommendations remain controversial for antenatal hydronephrosis with regard to diagnostic criteria, fetal interventions, risk classification, and postnatal diagnostic and therapeutic management (7, 10). Ultrasound is so far the best for the diagnosis and follow-up of both prenatal and postnatal hydronephrosis. The Onen hydronephrosis-grading system has been developed and upgraded based on evidence. It uses the quality of the renal parenchyma in combination of both affected and contralateral kidney size, including longitudinal length and atrophy. According to the recommendation, children with UTD Onen 1 or 2 need neither invasive investigation nor surgical treatment or antibiotics due to their benign outcome (10). In this study, the resolution was reported in 73% infants with transient UTD during the 6-month follow-up post birth. The 15 cases of UPJO were initially diagnosed of UTD Onen grade 2. The 7 cases of VUR were initially diagnosed with UTD Onen grade 1. The diagnosis of VUR in seven cases was identified due to episodes of UTI and postnatal UTD. It has not been established for an efficient way to screen for VUR (1). A study of 115 neonates demonstrated that most infants with prenatally assumed CAKUT had a favorable outcome during long-term follow-up, in particular infants with prenatal isolated unilateral or bilateral hydronephrosis (21). Oligohydramnios and postnatal bilateral renal anomalies were two predictors of non-favorable outcomes, i.e., the need for surgery and/or persistent defects with impaired renal function (21, 22). More cohort studies should be conducted to gain more knowledge on the long-term prognosis of UTD.

Several limitations of our study should be noted. First, the risk factors associated with CAKUT during the fetal stage may be missed in the situation of miscarriage, termination of pregnancy, stillbirth, or death of the fetuses prenatally diagnosed with renal anomalies. The proportion of births attended by skilled personnel has been above 90% in China since 2010. In Zhongshan City, there was no recode of homebirth during the study. Second, given that a high proportion of young couples are extremely mobile and difficult to contact, it is a significant accomplishment for this study to achieve a high retention rate of 98% for secondary and tertiary screening or further postnatal examinations. Nevertheless, approximately 40% of the study population was excluded from the risk factor analysis because of the incomplete records of medical charts of pregnancy. Most of the cases with a specific diagnosis of CAKUT were involved in the risk factor analysis. Only one case with a specific diagnosis of CAKUT was not enrolled in the risk factor analysis. Therefore, selection bias remains a concern for the risk factor analysis. However, the medical charts recorded by the medical staff were more reliable than questionnaires from the family members. Participation rates in epidemiologic studies have declined during the past years. In another study of birth cohort, a low response rate of questionnaire was presented, with only 50–70% (23). Third, the risk factors we evaluated excluded many other potential risk factors such as nutrition status during pregnancy. A positive association between maternal diabetes, obesity, smoking, or alcohol use and CAKUT in offspring has been reported (22–24). More detailed prenatal information and CAKUT phenotypes should be analyzed in further studies. Fourth, it is possible that some children with CAKUT were missed, as infants with normal ultrasound results and no clinical symptoms during the first 6 months postnatally were classified as normal. As clinical experience shows that most clinically relevant cases of CAKUT present with symptoms during the first 6 months of life, this period was chosen to minimize the number of cases lost to follow-up (15). Finally, further assessment of the cost-effectiveness of postnatal ultrasound screening for CAKUT should be conducted such as calculating a benefit-to-cost ratio based on long-term follow-up.

In conclusion, our study provided evidence that the prenatal and perinatal factors of male gender, preterm birth, antenatal abnormal ultrasound findings, gestational hypothyroidism, and oligohydramnios increase the risk of CAKUT in general. Postnatal ultrasound screening in high-risk infants is of great significance for the early detection of CAKUT. More epidemiological research should focus on potential factors with CAKUT to delineate the etiology of the individual conditions and to allow us to improve prenatal counseling.

## Data Availability Statement

The original contributions presented in the study are included in the article/Supplementary Material, further inquiries can be directed to the corresponding author/s.

## Ethics Statement

The studies involving human participants were reviewed and approved by the Ethics Committee of Zhongshan Boai Hospital, Southern Medical University KY-2019-002-17. Written informed consent to participate in this study was provided by the participants' legal guardian/next of kin.

## Author Contributions

JR, YL, and SF designed and supervised the study and critically revised the manuscript. HS and JR wrote the manuscript. XY, XX, XP, XL, ZS, and YL performed clinical examinations, collected medical data, and wrote the clinical part of the manuscript. XY, HS, and JR performed statistical analysis. All authors contributed to the clinical information analysis.

## Funding

JR was supported by a grant from the National Natural Science Foundation of China (NSFC-8182207), a grant from the Program of Shanghai Academic/Technology Research Leader (SHDC12016107), and a grant from Clinical Research Plan of SHDC (No. SHDC2020CR2064B). SF and YL were supported by a grant from Zhongshan social public welfare science and technology research project (No. 2019B1010).

## Conflict of Interest

The authors declare that the research was conducted in the absence of any commercial or financial relationships that could be construed as a potential conflict of interest.

## Publisher's Note

All claims expressed in this article are solely those of the author and do not necessarily represent those of their affiliated organizations, or those of the publisher, the editors and the reviewers. Any product that may be evaluated in this article, or claim that may be made by its manufacturer, is not guaranteed or endorsed by the publisher.
