# Supplementary material for: Risk Factors Associated With Renal and Urinary Tract Anomalies Delineated by an Ultrasound Screening Program in Infants
Source: Front Pediatr. 2022 Jan 24;9:728548. doi: 10.3389/fped.2021.728548 (PMC8819178; doi:10.3389/fped.2021.728548)
Supplement: Supplementary file 1 [file Data_Sheet_1.docx]

**Supplementary Table 1.** Number urinary tract dilation diagnosed post initial ultrasound screening

| Gender | Male | | | Female | | | Total (%) |
| --- | --- | --- | --- | --- | --- | --- | --- |
| Location | Left | Right | Bilateral | Left | Right | Bilateral |  |
| Onen-1 | 100 | 20 | 32 | 51 | 13 | 16 | 232 (93.2) |
| < 6 mm | 89 | 15 | 32 | 48 | 13 | 16 | 213 (85.5) |
| 6-10 mm | 11 | 5 | 0 | 3 | 0 | 0 | 19 (7.6) |
| Onen-2 | 10 | 1 | 1 | 4 | 0 | 0 | 16 (6.5) |
| Total | 110 | 21 | 33 | 55 | 13 | 16 | 248 |

**Supplementary Table 2.** Follow-up of ultrasound scanning in the 248 participants with abnormal findings at primary ultrasound screening

| **Gender** | | **Male** | | | | | | | | **Female** | | | | | | | | | | **Total (%)** | |  |  |
| --- | --- | --- | --- | --- | --- | --- | --- | --- | --- | --- | --- | --- | --- | --- | --- | --- | --- | --- | --- | --- | --- | --- | --- |
| **Degree** | | **Mild** | | **Moderate** | **severe** | | | | **Mild** | | | | **Moderate** | | | **severe** | | |  | | |  |  |
| **the secondary screening at the 3^rd^-month** | | | | | | | | | | | | | | | | | | | | | |  |  |
| Complete remission | | | 73 | | 1 | 2 | | 54 | | | | 2 | | | 0 | | | 132(53.2) | | | |  |  |
| Partial remission | | | 15 | | 8 | 7 | | 5 | | | | 0 | | | 2 | | | 37(14.9) | | | |  |  |
| stable | | | 34 | | 6 | 2 | | 16 | | | | 1 | | | 2 | | | 61(24.6) | | | |  |  |
| Progressive  UTI | | | 14  4 | | 1  0 | 1  2 | | 2  1 | | | | 0  0 | | | 0  0 | | | 18(7.26)  7* | | | |  |  |
| **the tertiary screening at 6^th^-month follow-up** | | | | | | | | | | | | | | | | | | | | | | | |
| Complete remission | | 104 | | 4 | | | 0 | | 70 | | | | 2 | | | 0 | | | 180(72.6) | | | | |
| Partial remission | | 2 | | 5 | | | 10 | | 1 | | | | 0 | | | 2 | | | 20(8.06) | | | | |
| stable | | 27 | | 7 | | | 2 | | 5 | | | | 1 | | | 2 | | | 44(17.7) | | | | |
| Progressive  UTI | | 3  8 | | 0  0 | | | 0  2 | | 1  4 | | | | 0  0 | | | 0  1 | | | 4(1.61)  15** | | | | |
